# Involvement of *Escherichia coli* DNA Replication Proteins in Phage Lambda Red-Mediated Homologous Recombination

**DOI:** 10.1371/journal.pone.0067440

**Published:** 2013-06-19

**Authors:** Anthony R. Poteete

**Affiliations:** Department of Microbiology and Physiological Systems, University of Massachusetts Medical School, Worcester, Massachusetts, United States of America; Florida International University, United States of America

## Abstract

The Red recombination system of bacteriophage lambda is widely used for genetic engineering because of its ability to promote recombination between bacterial chromosomes or plasmids and linear DNA species introduced by electroporation. The process is known to be intimately tied to replication, but the cellular functions which participate with Red in this process are largely unknown. Here two such functions are identified: the GrpE-DnaK-DnaJ chaperone system, and DNA polymerase I. Mutations in either function are found to decrease the efficiency of Red recombination. *grpE* and *dnaJ* mutations which greatly decrease Red recombination with electroporated DNA species have only small effects on Red-mediated transduction. This recombination event specificity suggests that the involvement of GrpE-DnaJ-DnaK is not simply an effect on Red structure or stability.

## Introduction

The Red homologous recombination system of bacteriophage λ operates via at least two distinct pathways [1]. One, called the strand invasion pathway, is RecA-dependent and replication-independent. The other, called the strand annealing pathway, is RecA-independent and replication-dependent. These pathways were initially characterized by Stahl and coworkers in studies of recombination between phage chromosomes in infected cells (see [2] for a review).

The strand invasion pathway is relatively well-defined, and closely integrated with the host’s homologous recombination system. In the absence of other λ proteins, the *red* gene products Redβ and λExo can functionally replace the RecBCD recombinase in both repair and recombination [3] [4]. In the Red-substituted *Escherichia coli* strain, recombination between chromosomes brought together by the natural processes of conjugation or phage infection depends on double strand breaks, RecA, and RecFOR [5]. In this respect, it resembles recombination between non-replicating λ chromosomes in infected cells, although, in the latter case, RecFOR is not required because the phage also encodes a protein, Orf, which can substitute [6] [7].

The RecA-independent, replication-dependent Red pathway is relatively less characterized. In Red-substituted *E. coli* lacking RecA, a non-replicating dsDNA phage chromosome introduced by infection recombines efficiently with an indigenous homology-bearing plasmid, if the plasmid is replicating and the non-replicating partner has a double strand break. The topology and kinetics of this recombination event suggest that the role of Red is to pair a ssDNA end with exposed ssDNA in a replication fork, and to induce a template switch, diverting the replisome onto the invading DNA [8].

Red-substituted *E. coli* also gains the ability to incorporate into its chromosome, or plasmids, homology-bearing linear dsDNA species, or oligonucleotides, introduced into the cell by electroporation [4] [9] [10]. If the targeting homologous sequences are short, on the order of 30–50 bp, incorporation is only mildly RecA-dependent. Research has suggested that in this event, called recombineering, electroporated dsDNAs are first converted to single strands by λExo, and then, like oligonucleotides, annealed by Redβ to ssDNA exposed by replication, preferentially on the lagging strand [11] [12]. Involvement of host DNA replication functions in recombineering has been explored to increase the efficiency of the process; one such enhancement, for example, can be obtained by mutation of *dnaG* to slow lagging strand initiation, exposing longer ssDNA regions for annealing [13].

In a search for Red-interacting DNA replication proteins in *E. coli*, it was found that Redβ bearing a C-terminal SPA affinity tag [14] inhibits cell growth. Mutants resistant to Redβ-SPA were isolated. Three bore mutations altering key functional residues in the GreA protein. In cells with normal Red, these mutations stimulated replication-independent recombination, but inhibited replication-dependent recombination [15].

In this study, involvement in Red recombination of two other host functions is examined. One, the GrpE-DnaK-DnaJ chaperone system, was uncovered by characterization of additional Redβ-SPA resistant mutants. The other, DNA Polymerase I (Pol I), was suggested by the early observation that λ *red* mutants fail to form plaques on an *E. coli polA1* mutant (Noreen Murray, cited in [16]).

## Materials and Methods

### F’ plasmids

F’ *polA* [17] consists of a 5-kb HindIII fragment bearing the *polA* gene and the chloramphenicol resistance-conferring *cat* gene from Tn9, inserted into the unique HindIII site of pOX38, which consists of the largest HindIII fragment of the plasmid F [18]. Red-mediated recombination was used to replace the *cat* gene with *tetRA* from Tn10, generating F’ *polA-tet.* The entire insert was then replaced sequentially by *cat* and by *tetRA*, generating F’ *cat* and F’ *tet*. F’ *grpE-cat* was constructed by using Red-mediated recombination to insert the *cat* gene 32 bp downstream from *grpE* in the chromosome, then replacing *tetRA* in F’ *tet* with *grpE-cat*; the *cat* gene was subsequently replaced by *tetRA*, generating F’ *grpE-tet*.

### Bacterial strain construction

Strains described in this study are all derivatives of MG1655, although some of the mutant alleles were constructed in the reduced-genome MG1655 derivative MDS12 [19]. Mutations in the *grpE* ribosome-binding site and coding sequence were installed by the use of Red-mediated recombination in two steps. The sequences to be altered were replaced with the *cat* gene, with selection for chloramphenicol resistance; the *cat* gene was subsequently replaced by the altered sequence, with ampicillin or kanamycin enrichment for chloramphenicol-sensitive recombinants, as described previously [15]. Because the *grpE* gene is essential [20], [21], these steps were carried out in strains bearing F’ *grpE-tet*. Chromsomal *cat* insertions were distinguished from F’ insertions by their inability to be transferred via conjugation to a chloramphenicol-sensitve recipient. The chromosomal *grpE* mutations were subsequently transferred to non F-containing cells by P1 transduction, employing linkage to *tyrA*. Because of the limited viability and Red recombination of strains bearing *polA* mutations, *polA* mutants were constructed in the same way as *grpE* mutants, in strains bearing F’ *polA-tet*, and transferred by P1 transduction via linkage to *metE*.

Because of the poor growth of phage P1 on strains bearing *dnaK* or *dnaJ* mutations [22], deletion alleles bearing an FRT-flanked kanamycin resistance element [21] were PCR-amplified and introduced into strains by the use of Red-mediated recombination. The kanamycin-resistance gene was subsequently removed by the use of the FLP-bearing plasmid pCP20 [23].

Red-substituted *E. coli* strains in this study have *Ptac-gam-bet-exo* in place of r*ecC-ptr-recB-recD*. The substitution, designated *recBCDΔred*, was made by Red-mediated, oligonucleotide-directed deletion of *cat* from the previously described *Ptac-gam-bet-exo-cat* substitution of strain KM32 [24].

Other *E. coli* genes were deleted from the chromosome by Red-mediated replacement with *cat* and subsequent deletion of *cat*. The deletion alleles were transferred by P1 transduction via linkage with auxotrophic markers, in the following combinations: *polBΔ – leuA, dinBΔ – proBA, umuDCΔ – icd, recDΔ* (or *recBCDΔred*) *– argA.*

### Red tests

Strains to be tested were grown by inoculation of LB medium with single colonies, followed by overnight incubation, standing at 30°C. Cultures were diluted 14- to 21-fold into 20 ml LB supplemented with 1 mM isopropylthiogalactopyranoside (IPTG) to induce *red* expression, and grown with aeration at 30°C to an A_600_ of 0.2, corresponding to a cell count of 6–8×10^7^/ml for TP1369 (*recBCDΔred*, otherwise wild type). Cultures were chilled in an ice-water bath, and cells were collected by centrifugation in the cold, resuspended with 0.85 ml water, and transferred to microcentrifuge tubes. Cells in the microfuge tubes were pelleted in the cold, resuspended with 1 ml water, pelleted again, and finally resuspended with 100 µl water. 5 µl of a mixture containing approximately 100 ng of a 906-bp dsDNA *cat* cassette targeted to the *lacZ* gene, 10 ng of a 70-base oligodeoxyribonucleotide designed to introduce streptomycin resistance-conferring mutations into the *rpsL* gene, and 0.01 ng of an intact kanamycin resistance-conferring plasmid was added to 50 µl samples of the cells, which were subjected to electroporation, followed by dilution into 12 ml LB medium, and standing incubation for 22–24 hr at 30°C to permit expression of antibiotic resistance. Control electroporation mixtures contained water in place of the DNA mixture. The cultures were plated at 30°C on LB, or LB supplemented with antibiotics (chloramphenicol at 15 µg/ml, streptomycin at 100 µg/ml, or kanamycin at 40 µg/ml) to determine titers. Results are reported as ratios of chloramphenicol-resistant or streptomycin-resistant recombinants to kanamycin-resistant transformants, normalized to the ratio determined for wild type cells in the same experiment.

The dsDNA *lacZ*-targeting cat cassette was synthesized by PCR amplification of the Tn9 *cat* gene with primers containing 40 base flanks,

cat47

GATGTGGATTGGCGATAAAAAACAACTGCTGACGCCGCTGATGAGACGTTGATCGGCACG and

cat48

CTTCACTTACGCCAATGTCGTTATCCAGCGGTGCACGGGTATTCAGGCGTAGCACCAGGC

The *rpsL*-targeting oligonucleotide was designed to pair with the lagging strand [10], and introduce the mutation Lys87–>Arg [25] with multiple mismatches (underlined) chosen according to Sawitzke et al. [26] to evade mismatch repair.

Str02

ACGTACGGTGTGGTAACGAACACCCGGGAGGTCACGTACACGACCGCCACGGATCAGGATCACGGAGTGC

The plasmid used in these experiments, pTP1125, consists of the *polA*-independent pSC101 replicon [27] and the *aph* gene of Tn903.

Strains to be tested for relative ability to form recombinants via P1 transduction were grown by inoculation of LB medium with single colonies, followed by overnight incubation, standing at 30°C. Cells were pelleted by centrifugation in the cold, and resuspended on ice with a mixture of 0.2 vol LB plus 0.1 vol (30 mM MgSO_4_, 15 mM CaCl_2_); 0.6 ml of the resuspended cells was mixed with enough of a P1 vir lysate grown on a *bioAΔtet* donor strain to produce about 200 transductants in the wild type recipient. Incubation was for 45 min at 30°C, followed by pelleting in a microcentrifuge, resuspension with a drop of water, and plating at 30°C on LB supplemented with tetracycline at 15 µg/ml.

### λ strains

The *γ210* mutation is an amber mutation in the *gam* gene [15], [16]. The *b1453* deletion removes sequences from *att* through *gam* [28]. A simple deletion, *redΔ*, removing sequences between the first three codons of *redβ* (*bet*) and the last three codons of *redα* (*exo*) was constructed in two steps, employing Red-mediated recombination as described by Oppenheim et al. [29]. The sequences were first replaced by a cassette consisting of the *cat* gene and a site for the I-SceI endonuclease. Recombinants were selected for the ability to form chloramphenicol-resistant lysogens. The cassette was subsequently deleted by recombination with an oligonucleotide, and the recombinant was identified by plating on a strain bearing an I-SceI expressing plasmid, which reduces the efficiency of plating and plaque size of λ bearing an I-SceI site. Phages used for plating tests bear the temperature-sensitive *cI857* repressor mutation. M9 minimal medium with 0.2% maltose was inoculated with single colonies of the bacterial strains to be tested, and incubated standing for 22–26 hr at 37°C. Portions of the cultures (0.2 – 0.3 ml) were mixed with 2.5 ml molten soft agar (1% tryptone, 0.25% NaCl, 0.7% agar), and spread on agar plates of the same composition but 1.3% agar. Dilutions of phage stocks were spotted on the surface, and plates were incubated overnight at 37°C.

## Results

### GrpE-DnaK-DnaJ

**Figure 1:**
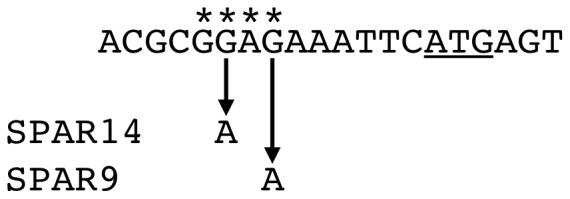
Sequences of *grpE* ribosome-binding site mutations. Asterisks indicate bases complementary to the 3’ end of 16S rRNA. The initiation codon is underlined.

Redβ-SPA resistant mutants were isolated from cultures subjected to mutagenesis with *N*-methyl-*N’*-nitro-*N*-nitrosoguanidine [15]. Two such mutants, designated SPAR14 and SPAR9, were mapped and sequenced. Initial tests established linkage between SPAR14 and a transposon insertion in *norV*; and between SPAR9 and a transposon insertion in *yfiF*. P1 transductional crosses established close linkage of both mutations to *tyrA*, with the order *yfiF-tyrA-SPAR*; co-transduction frequencies suggested that both mutations were either in or near *grpE*. Sequencing revealed that each bore a single base pair substitution in the ribosome-binding site of *grpE* (Figure 1). GrpE is the nucleotide exchange factor for the major DnaJK protein folding machine of *E. coli* [30]. For further study, SPAR14 and SPAR9 were introduced by means of Red-mediated recombination into unmutagenized *E. coli* MG1655; for tests of Red function, the *recBCDΔred* substitution was introduced as well.

The SPAR14 and SPAR9 substitutions reduce the complementarity of *grpE* mRNA’s upstream sequence to the 3’ end of 16S rRNA, and so would be predicted to reduce *grpE* expression. Consistent with this hypothesis, expression of additional *grpE* from F’ *grpE-tet* restores Redβ-SPA sensitivity to the mutants (Table 1). In a test of Red-mediated recombination between the *E. coli* chromosome and a dsDNA species introduced by electroporation, both mutants exhibited mild defects, with SPAR14 decreasing recombination slightly more than SPAR9 (Table 2). The mutations had little or no effect on recombination with an oligonucleotide.

**Table 1:** Sensitivity to Redβ-SPA

Strain	Chromosomal genotype	Plasmid(s)	Growth with no induction^a^	Growth with induction (IPTG)^a^
MG1655	wild type	pTP1206^b^	+	–
TP1534	*SPAR14*	pTP1206	+	+
TP1535	*SPAR9*	pTP1206	+	+ (weak)
TP1549	wild type	pTP1206 + F’ grpE-tet	+	–
TP1551	*SPAR14*	pTP1206 + F’ grpE-tet	+	–
TP1553	*SPAR9*	pTP1206 + F’ grpE-tet	+	–
TP1548	wild type	pTP1206 + F’-tet	+	–
TP1550	*SPAR14*	pTP1206 + F’-tet	+	+
TP1552	*SPAR9*	pTP1206 + F’-tet	+	+ (weak)

a. Growth of patches made from individual colonies on LB plates supplemented with kanamycin at 20 µg/ml, plus or minus 1 mM IPTG, at 30°C. Plates for comparison of F’-bearing strains were additionally supplemented with tetracycline at 15 µg/ml.

b. pTP1206 confers resistance to kanamycin and synthesizes Redβ-SPA under control of P_tac_ and the Lac repressor [15].

**Table 2:** Effects of GrpE-DnaK-DnaJ system mutations on Red-mediated recombination.

Strain	Relevant genotype	Electroporated dsDNA cassette^a^	Electroporated oligonucleotide^a^	P1 transduced chromosomal marker^b^
TP1369	wild type^c^	1	1	1
TP1544	*SPAR14*	0.18±0.04	0.90±0.08	
TP1545	*SPAR9*	0.36±0.14	0.89±0.05	
TP1594	*grpE-G122D*	0.012±0.006	0.076±0.016	0.47±0.07
TP1621	*grpE-G122D/F’ tet*	0.011±0.006	0.079±0.004	
TP1622	*grpE-G122D/F’ grpE-tet*	0.42±0.17	0.90±0.18	
TP1602	*dnaKΔFRT*	nrd^d^	nrd^d^	0.03±0.01
TP1603	*dnaJΔFRT*	nrd^d^	0.005±0.003	0.40±0.01
TP1625	*redΔ*	nrd^d^	0.006±0.001	0.01±0.002

a. Numbers represent the ratios of recombinants to transformants, normalized to the ratio observed for the wild type strain in the same experiment. Means and standard errors for three experiments are shown. The absolute ratio of chloramphenicol-resistant recombinants (dsDNA cassette) to kanamycin-resistant transformants for wild type had a mean value of 0.078 and a standard deviation of 0.038 in 11 experiments; the corresponding figures for streptomycin-resistant recombinants (oligonucleotide) were 0.25 and 0.08, respectively.

b. Numbers represent numbers of recombinants recovered, normalized to the number recovered with a wild type recipient in the same experiment. Means and standard errors for three experiments are shown.

c. All strains bear the *recBCDΔred* substitution, except for TP1625, in which *recBCD* is deleted without any substitution.

d. nrd  =  no recombinants detected.

The *grpE* gene was originally identified by the isolation of a mutant in which wild type λ could not replicate, but certain λ gene P mutants could [31]. The mutation in this strain, *grpE280*, changes residue 122 in the encoded protein from glycine to aspartic acid [32]. To test further the involvement of GrpE in Red recombination, this mutation (G122D) was introduced into *grpE* in the MG1655 chromosome, along with the *recBCDΔred* substitution. As shown in Table 2, G122D reduces Red-mediated recombination with an electroporated oligonucleotide 12-fold, and with a dsDNA cassette 80-fold. This phenotype is mostly recessive: addition of F’ *grpE-tet* to the cell restores recombination to nearly the wild type level. In contrast to its strong effect on Red-mediated recombination with electroporated dsDNA, the G122D mutation reduces the efficiency of Red-mediated P1 transduction of a chromosomal marker only 2-fold.

GrpE is part of the GrpE-DnaK-DnaJ chaperone system, which is required for *E. coli* replication at high temperatures, and for λ replication at all temperatures [33], [34]. The observation that some forms of Red recombination are dependent upon GrpE therefore suggests possible involvement of DnaK and DnaJ as well. Deletion of *dnaJ* indeed reduces Red-mediated recombination with an electroporated oligonucleotide 200-fold, and with a dsDNA cassette below detectable levels in the test employed (Table 2). Like the *grpE-G122D* mutation, *dnaJΔFRT* also reduces Red-dependent P1 transduction only slightly. Possible involvement of DnaK is more difficult to ascertain. Deletion of *dnaK* also reduces Red-mediated recombination, but quantitation of the effect is difficult because of pleiotropic effects of the mutation decreasing viability and transformability (not shown). The *dnaK* deletion additionally exhibitis a 30-fold reduction in Red-dependent transduction efficiency, but it is not clear whether this effect is on recombination or on some other aspect of infection by P1 transducing particles. The Red-dependency of recombination in these assays is shown by the fact that deletion of *red* strongly reduces recombination with DNA species introduced into the cell by either electroporation or transduction (Table 2).

### Pol I

**Figure 2:**
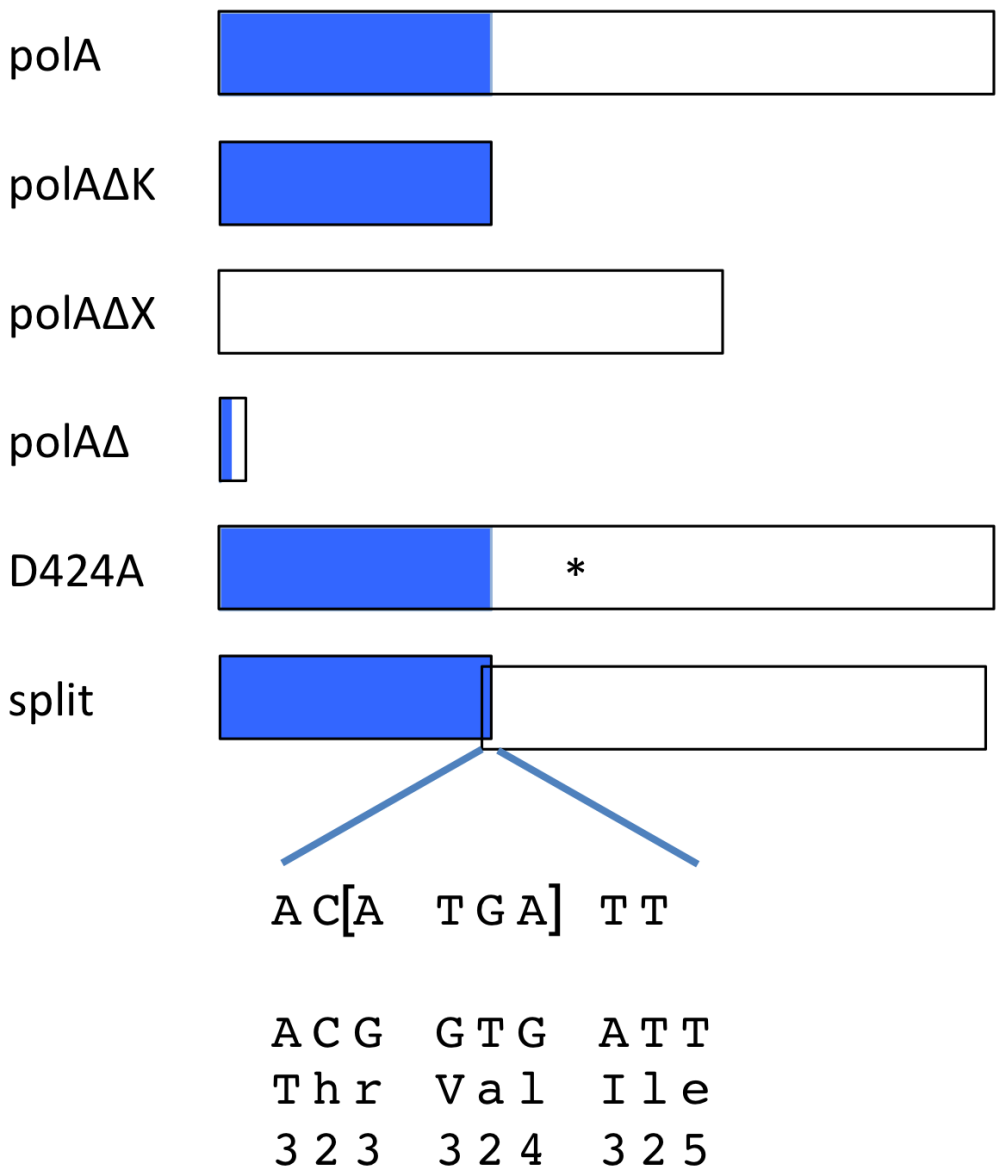
Chromosomal mutants altering Pol I constructed for this study. Blue and white segments indicate the N-terminal domain, consisting of residues 1–323 and harboring the 5’-to-3’ exonuclease; and the C-terminal domain, consisting residues 324–928 and harboring the 3’-to-5’ exonuclease and DNA polymerase, respectively. The sequence of the *polA-split* mutation is indicated below the map; below that is the relevant segment of the wild type sequence.

The *E. coli polA* gene, which encodes Pol I, is not strictly essential—a strain lacking it can grow slowly on minimal medium, but exhibits marginal or no viability on rich medium, depending on the strain background [17] [21]. A simple chromosomal *polA* deletion mutant of *E. coli* strain MG1655 constructed for this study exhibits these properties (not shown). To distinguish between effects on growth and effects on recombination, and because electroporation-based tests of Red activity cannot be done with cells grown on minimal medium [26], chromosomal *polA* mutants with alterations more subtle than complete deletion were also constructed (Figure 2). Alterations were guided by understanding of PolA based on extensive studies of its structure and function by many groups of investigators [35] [36].

Three types of alteration were introduced into *polA*. The first type eliminates one domain at a time. In the *polAΔK* mutant, the C-terminal domain-encoding segment of the gene is deleted, leaving only codons 1-323. This remnant corresponds to the stable N-terminal fragment of Pol I, which retains the 5’-to-3’ exonuclease activity of the intact protein [37]. Phenotypically, *polAΔK* is predicted to mimic the original *polA1* mutation, a nonsense mutation stopping translation of the C-terminal domain [38] [39]. In the *polAΔX* mutant, codons 2-323 are deleted, leaving a partial gene equivalent to the Klenow fragment gene of Joyce and coworkers [40], but fused to the normal *polA* upstream sequences, and located in the chromosome. The mutant variant retains the polymerase and 3’-to-5’ exonuclease activities of the intact protein. The second type of alteration precisely neutralizes key individual catalytic residues. Single codon substitutions D140A, D424A, and D705A were placed in the chromosome. These mutations eliminate the 5’-to-3’ exonuclease, 3’-to-5’ exonuclease, and the DNA polymerase activities, respectively [37], [41], [42]. However, of the three substitutions, only D424A could be transduced from the partially diploid F’ *polA-tet* containing strain in which it was constructed to a normal haploid recipient. Apparently the other two are lethal mutations in MG1655. The lethality of D705A has been observed previously [42]. A third type of alteration, *polA-split*, was engineered to produce the N- and C-terminal domains as separate proteins. The rationale was based on the finding that the phenotype of the mutant *polA12* enzyme apparently results from a defect in the coordination of polymerase and 5’-to-3’ exonuclease activities [43]. The mutation constructed for this study, a replacement of two G-C basepairs with one A-T, introduces a frameshift which makes codon 324 a TGA stop codon, overlapping a new ATG initiation codon for a polypeptide corresponding to the Klenow fragment in *polAΔX*.

The effects of the *polA* mutations on Red-mediated recombination between the chromosome and electroporated DNA species are summarized in Table 3. Both *polAΔK* and *polAΔX* mutations substantially reduce the yield of recombinants produced by recombination with either a dsDNA cassette or an oligonucleotide. However, the low yields are largely due to lower cell densities and lower viabilities in the mutant cultures. After controlling for these factors by normalizing to the yield of transformants with an intact plasmid in the same electroporation mixture, neither mutation appears to have a strong effect on recombination specifically, though the *polAΔX* mutation does decrease recombination with the dsDNA casette approximately 6-fold. The *polA-split* mutation, on the other hand, has little effect on viability, but a stronger effect on recombination with the dsDNA cassette, reducing it approximately 25-fold; its effect on recombination with the oligonucleotide is less than 2-fold. The D424A mutation has little or no effect in these tests.

**Table 3:** Effects of DNA polymerase mutations on Red-mediated recombination^a^.

Strain	Relevant genotype	Electroporated dsDNA cassette	Electroporated oligonucleotide
TP1369	wild type^b^	1	1
TP1584	*polAΔK*	0.62±0.32	1.95±1.53
TP1585	*polAΔX*	0.16±0.10	1.30±0.60
TP1611	*polA-split*	0.04±0.01	0.59±0.09
TP1612	*polA-D424A*	0.44±0.11	1.09 ±0.12
TP1583	*polBΔ dinBΔ umuDCΔ*	0.52±0.06	1.03±0.24
TP1618	*polBΔ dinBΔ umuDCΔ polAΔK*	0.06±0.02	0.67±0.30

a. Numbers represent the ratios of recombinants to transformants, normalized to the ratio observed for the wild type strain in the same experiment. Means and standard errors for 3 – 7 experiments are shown. Absolute values of the wild type ratios are given in the notes to Table 2.

b. All strains bear the *recBCDΔred* substitution.

In addition to the replication/repair DNA polymerases Pol I and Pol III, *E. coli* harbors three additional DNA polymerases, Pol II, Pol IV, and Pol V. These polymerases are collectively described by their shared property of catalyzing translesion synthesis (TLS) on damaged templates (for a review, see [44]). Possible involvement of the TLS polymerases in Red-mediated recombination was tested by deleting their respective genes, *polB*, *dinB*, and *umuDC*, and assembling a triple mutant strain. Red-mediated recombination with electroporated DNA species in this strain is nearly as efficient as in wild type. Combining these three deletions with *polAΔK* creates a strain lacking all DNA polymerases except Pol III. The strain’s deficiency in Red-mediated recombination with a dsDNA is approximately 10-fold greater than that of the single *polAΔK* mutant.

To relate the effects of various *polA* mutations on Red-promoted recombination to the function of Red in the development of phage λ, plating of wild type and mutant λ phages on the DNA polymerase mutants was tested, and is summarized in Table 4. As expected, the strong null mutants *polAΔ*, *polAΔK*, and *polAΔX* fail to support plaque formation by λ lacking *red*, *gam,* or both *red* and *gam* functions (Feb phenotype [16]). The polA-split mutation exhibits a partial Feb phenotype: λ *redΔ* forms barely visible plaques at reduced efficiency, while λ *γ210* and λ *b1453* (deleted for both *red* and *gam*) make no plaques. The D424A mutation has no effect on λ plating; neither does the triple polymerase deletion *polBΔ dinBΔ umuDCΔ.* The quadruple polymerase deletion *polBΔ dinBΔ umuDCΔ polAΔK* exhibits the same phenotype as *polAΔK* alone.

**Table 4:** λ plaque formation on DNA polymerase and RecD mutants^a^.

Strain	Relevant genotype	λ *cI857*	λ *cI857 redΔ*	λ *cI857 γ210*	λ *cI857 b1453*
MG1655	wild type	++	++	+	+
TP1537	*polAΔ*	+	–	–	–
TP1506	*polAΔK*	+	–	–	–
TP1476	*polAΔX*	+	–	–	–
TP1564	*polA-split*	+	±	–	–
TP1565	*polA-D424A*	++	++	+	+
TP1503	*polBΔ dinBΔ umuDCΔ*	++	++	+	+
TP1556	*polBΔ dinBΔ umuDCΔ polAΔK*	+	–	–	–
TP1576	*recDΔ*	++	++	++	++
TP1588	*polAΔK recDΔ*	+	±	+	+
TP1589	*polAΔX recDΔ*	+	±	+	+

a. ++  =  large plaques, +  =  small plaques, ±  =  low efficiency of plating, barely visible plaques, –  =  no plaques.

Studies of λ replication (reviewed in [45]) indicated that Red was required for phage replication in the absence of Pol I. Studies done at that time did not distinguish between the two ways in which Red contributes to λ replication: by promoting recombination, and by preventing destruction of λ chromosomes by RecBCD, particularly in the absence of the λ Gam protein, which directly inhibits RecBCD (for a review, see [46]). To investigate this question further, the *polAΔK* and *polAΔX* mutations were combined with a deletion of *recD*, which selectively eliminates RecBCD’s nuclease activity. As shown in Table 4, knocking out *recD* partially suppresses the Feb phenotype: λ *γ210* and λ *b1453* both form plaques on the double mutants; λ *redΔ* grows better than it does on either *polAΔK* or *polAΔX* alone, making marginal plaques. As discussed below, this observation suggests that the host’s homologous recombination system can substitute for Red in supporting λ replication in a *polA* mutant.

## Discussion

These experiments point to the involvement of two host functions in Red-mediated recombination between the chromosome and electroporated DNA species. Mutations altering GrpE-DnaK-DnaJ or Pol I impair this activity. The nature of the involvement—direct or indirect—as well as the details of the mechanism, remain to be determined. However, extensive studies of both of these cellular functions provide some clues.

GrpE-DnaK-DnaJ is a chaperone system, with a role in maintaining the folded states of 409 or more *E. coli* proteins [47]. One might therefore guess that its role in Red recombination is indirect, contributing to the folding or stability of either Redβ or λExo or both. However, as shown in Table 2, certain mutations in *grpE* and *dnaJ* strongly depress Red-mediated recombination between the chromosome and electroporated DNA species, but have only mild effects on Red-mediated transduction. This specificity suggests that simple destabilization of Red protein(s) is not the reason for the recombination defects of the mutants, but rather, that GrpE-DnaK-DnaJ has a more specific role in promoting Red-mediated recombination with short electroporated DNA species. There is a precedent for hypothesizing a role for GrpE-DnaK-DnaJ that is more specific than promoting protein stability, in at least one other process. GrpE-DnaK-DnaJ promotes elongation in λ replication by destabilizing the interaction between the DnaB helicase and λ’s P protein, freeing DnaB from the origin complex, enabling it to unwind DNA [33].

Involvement of Pol I in Red recombination with short electroporated DNA species is unsurprising. This Red activity takes place at replication forks [10], so one might expect it to be affected by anything that affects DNA replication. Pol I is an important participant in normal replication; replication in its absence is aberrant in numerous ways [43]. It is additionally easy to imagine that the protein’s polymerase and/or exonuclease activities could be involved in processing of DNA strands annealed by Red to exposed template strands in the replication fork. The template switch model for Red recombination, on the other hand, suggests a more interesting role for Pol I: perhaps it is the polymerase which is most likely to be induced by Red to switch templates. Template-switching was first observed in studies of Pol I [48]. Results shown in Table 3 additionally suggest that some or all of the TLS DNA polymerases, Pols II, IV, and V, may have a secondary role in Red-mediated recombination, more readily observed in the absence of Pol I. While this paper was under review, Li et al. ([49]) reported that Pol I and Pol III both are directly involved in Red-mediated oligo recombination.

It has been known for many years that Red recombination in the absence of RecA requires replication [50], and that proper replication of phage λ in the absence of Pol I requires Red [45]. One question concerning this relationship that has not been answered is whether λ’s requirement in a *polA* mutant is for Red in particular (or a related phage recombination system), or just for any functioning homologous recombination system. The observations summarized in Table 4 favor the latter answer. In a *polA* mutant host infected with a λ *red* mutant, none of the three homologous recombination pathways which might be available—RecBCD-RecA, Red-RecA, or Red-replicative—is functional: Red is inactivated by mutation, and RecBCD is inactivated by Gam protein. Elimination of Gam by mutation does not help much, because uninhibited RecBCD promotes recombination of λ chromosomes only inefficiently [51]. However, in a *polA recD* double mutant, elimination of Gam is effective, because RecD-less RecBC is far more active in promoting recombination of λ chromosomes than intact RecBCD [51]. Thus, the λ *red gam* double mutant is able to form plaques on a *polA recD* double mutant, because RecBC-RecA can substitute for the Red system in supporting phage replication.
